# Post‐COVID‐19‐associated multiorgan complications or “long COVID” with literature review and management strategy discussion: A meta‐analysis

**DOI:** 10.1002/hsr2.1211

**Published:** 2023-04-14

**Authors:** Phool Iqbal, Fateen Ata, Hassan Chaudhry, Bassam Muthanna, Hafiz Waqas Younas, Syed Ata ul Munamm, Rohit Sharma, Kahtan Fadah, Shereen Elazzazy, Anas Hamad, Osama Said Abu Tabar, Nabil E. Omar

**Affiliations:** ^1^ Department of Internal Medicine New York Medical College/Metropolitan Hospital Center New York New York USA; ^2^ Department of Endocrinology Hamad Medical Corporation Doha Qatar; ^3^ Department of Respiratory Medicine University Hospital of Leicester Leicester UK; ^4^ Department of Geriatrics University of Illinois at Chicago Chicago Illinois USA; ^5^ NHS Lanarkshire Leicester UK; ^6^ Department of Public Health Health Services Academy Islamabad Islamabad Pakistan; ^7^ Department of Internal Medicine Geisinger Health System Danville Pennsylvania USA; ^8^ Department of Internal Medicine Texas Tech University Health Sciences Center El Paso Lubbock Texas USA; ^9^ Pharmacy Department National Centre for Cancer Care and Research, Hamad Medical Corporation Doha Qatar; ^10^ Department of Clinical Pharmacology National Centre for Cancer Care and Research, Hamad Medical Corporation Doha Qatar; ^11^ Cleveland Clinic Abu Dhabi, Clinical Assistant Professor of Medicine, Cleveland Clinic Lerner College of Medicine Case Western Reserve University Abu Dhabi UAE

**Keywords:** COVID‐19 complications, long COVID, Meta‐analysis, Post COVID‐19, SARS‐CoV‐2 sequelae

## Abstract

**Objective:**

To investigate the post‐COVID‐19 long‐term complications or long COVID of various organ systems in patients after 3 months of the infection, specifically before the Omicron variant, with comparative literature analysis.

**Methods:**

A systemic literature search and meta‐analysis were conducted using multiple electronic databases (PubMed, Scopus, Cochrane library) with predefined search terms to identify eligible articles. Eligible studies reported long‐term complications of COVID‐19 infection before the Omicron variant infection. Case reports, case series, observational studies with cross‐sectional or prospective research design, case–control studies, and experimental studies that reported post‐COVID‐19 complications were included. The complications reported after 3 months after the recovery from COVID‐19 infection were included in the study.

**Results:**

The total number of studies available for analysis was 34. The effect size (ES) for neurological complications was 29% with 95% confidence interval (CI): 19%–39%. ES for psychiatric complications was 24% with 95% CI: 7%–41%. ES was 9% for cardiac outcomes, with a 95% CI of 1%–18%. ES was 22%, 95% CI: 5%–39% for the gastrointestinal outcome. ES for musculoskeletal symptoms was 18% with 95% CI: 9%–28%. ES for pulmonary complications was 28% with 95% CI: 18%–37%. ES for dermatological complications was 25%, with a 95% CI of 23%–26%. ES for endocrine outcomes was 8%, with a 95% CI of 8%–9%. ES size for renal outcomes was 3% with a 95% CI of 1%–7%. At the same time, other miscellaneous uncategorized outcomes had ES of 39% with 95% CI of 21%–57%. Apart from analyzing COVID‐19 systemic complications outcomes, the ES for hospitalization and intensive care unit admissions were found to be 4%, 95% CI: 0%–7%, and 11% with 95% CI: 8%–14%.

**Conclusion:**

By acquiring the data and statistically analyzing the post‐COVID‐19 complications during the prevalence of most virulent strains, this study has generated a different way of understanding COVID‐19 and its complications for better community health.

## 1 INTRODUCTION

World Health Organization (WHO) has developed a term for post‐COVID‐19 conditions as “long COVID,” defined as coronavirus symptoms that persist or return 3 months after a person becomes ill from infection SARS‐CoV‐2, commonly known as COVID‐19.^1^ The most common are headache, fatigue, insomnia, cognitive impairment, posttraumatic stress disorder (PTSD), loss of taste and smell, with heart and kidney problems as well.^1^

Since March 11, 2020, COVID‐19 has been declared a pandemic that has affected the medical community and economy worldwide. Moreover, till now, the world has been struggling to overcome the disease and its aftermath. Initially, many acute cases of COVID‐19 have overwhelmed healthcare resources worldwide, especially the intensive care unit (ICU) departments, and the recovered cases with post‐COVID‐19 complications have created a challenge for healthcare workers.^2^ Currently, long COVID is a situation of concern for a healthy community^3^ as estimates of the population that experience post‐COVID‐19 conditions are roughly 13.3% after 1 month of infection, 2.5% after 3 months or longer while more than 30% after 6 months in hospitalized patient, respectively.^4^

Highly infectious and virulent variants of COVID‐19, that is, Alpha (B.1.1.7), Beta (B.1.351), and Delta (B.1.617.2) and later its variant AY.4.2, sometimes known as delta plus were seen earlier in the course of the pandemic. COVID‐19 alpha variant was first reported in Great Britain, a Beta variant in South Africa, and a delta in India in the late 2020s.^5^ Later, these variants were also found in other parts of the world due to high infectivity rates. These variants are considered more contagious and virulent, with a greater risk of hospitalization and death.^5^ Moreover, this poses a higher risk of increased morbidity and mortality to the community.^5^

On the contrary, the COVID‐19 Omicron variant is considered relatively less virulent owing to its genetic makeup, mutation, and the increased number of vaccinations across the globe.^6^, ^7^, ^8^ COVID‐19 mainly affects the respiratory system and commonly presents as fever, cough, and upper respiratory tract infection (URTI). Shortness of breath but other unrelated clinical signs and symptoms such as decreased appetite, anosmia, diarrhea, electrolyte imbalance such as hyponatremia, acute renal failure, fatal thromboembolic complications like stroke, myocardial infarction, pulmonary embolism, and deep venous thrombosis are reported in the literature as well.^9^, ^10^, ^11^, ^12^, ^13^, ^14^ COVID‐19 has the propensity to cause multiorgan damage through various pathophysiological mechanisms, but the most prominent ones are the cytokine storm and dysregulated immunity.^15^ Many patients have recovered from the infection. However, COVID‐19 recovered cases have ongoing sequelae of the disease, including psychological complications such as anxiety, depression, insomnia, PTSD, normal daily functioning, and so on, thus causing ongoing utilization of health resources in dealing with its aftermath. The literature is being updated now and then by the scientific community to understand and overcome the chaotic situation caused by COVID‐19 sequelae. COVID‐19‐associated signs, symptoms, complications, and outcomes have been reported in various segregated studies, but most of the reported long COVID‐19 symptoms/complications were without highlighting strain prevalence. We aim to integrate post‐COVID‐19 complications after 3 months of the infection through our meta‐analysis until late 2021, when alpha, beta, and delta variants were prevalent.

## 2 METHODOLOGY

### 2.1 Search strategy, study selection, and data collection

A systemic literature search and meta‐analysis were conducted using multiple electronic databases (PubMed, Scopus, Cochrane library) with predefined search terms to identify eligible articles. Eligible studies reporting long‐term complications of COVID‐19 infection before the Omicron variant infection worldwide, including case reports, case series, observational studies with cross‐sectional or prospective research design, case–control studies, and experimental studies that reported post‐COVID‐19 complications, were included. The complications reported after 3 months after the recovery from COVID‐19 infection were included in the study. The following search term will be used: (“Post COVID”) OR (“Post Covid‐19”) OR (“Post coronavirus”) OR (“After COVID”) OR (“Post Severe acute respiratory syndrome coronavirus [SARS‐CoV‐2]”) OR (“After SARS‐CoV‐2”) AND (“Complications”) AND (“sequelae”). This protocol follows MOOSE Guidelines for Meta‐Analyses and Systematic Reviews of Observational Studies and the PRISMA‐P (Preferred Reporting Items for Systematic Reviews and Meta‐Analyses) guidelines.^16^, ^17^

### 2.2 Inclusion criteria and exclusions criteria

Eligible studies should report the complications of COVID‐19 infection in patients who recovered from the infection. All literature, including clinical trials, case reports, case series, and observational studies (retrospective and prospective) from any date till March 2021 in the English language, will be included. Studies in languages other than English will not be included. The study population will include pediatric (≤18 years) as well as adult (>18 years) patients who developed long‐term complications of COVID‐19 infection. The included patients should have a confirmed diagnosis of SARS‐CoV‐2 infection and should have a documented recovery confirmed with a negative reverse transcription polymerase chain reaction (RT‐PCR). Patients should have a complication in any system at least 3 months after the first positive RT‐PCR. Exclusion criteria include patients with COVID‐19 who did not have a positive RT‐PCR upon diagnosis, those who had complications during the infection (defined by a positive RT‐PCR), and those who had the onset of complications in less than 3 months of the positive RT‐PCR.

### 2.3 Quality assessment

**Figure 1:**
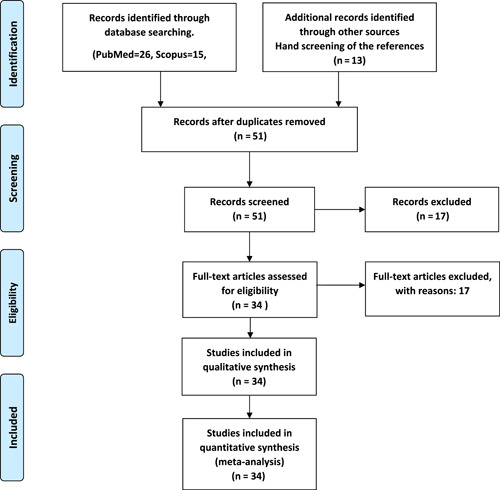
PRISMA 2009 flow diagram. From: Moher et al.^16^  (for more information, visit: www.prisma-statement.org).

Two reviewers independently evaluated study quality using the statistical methodology and categories guided by the Cochrane Collaboration Handbook and PRISMA guidelines (Figure 1). The protocol is registered at the International Prospective Register of Systematic Reviews (registration number: CRD42021240027).

### 2.4 Data synthesis and statistical analysis

Results were analyzed descriptively, and meta‐analysis was conducted to derive the pooled proportions and effect estimates. We used pooled proportional analysis using STATA software with the command “Metaprop.” The total number of studies was 34, with a total cohort of 247,209 patients. We excluded case reports and case series during the analysis because of concerns regarding the precision and potential bias of prevalence estimates. We compared studies with the number of patients having each outcome available. Random effects single‐arm meta‐analysis of proportions were performed. The pooled effect sizes (ESs) for post‐COVID‐19 systemic complications were represented as forest plots and included neurological, psychiatric, cardiac, gastrointestinal, musculoskeletal, pulmonary, dermatological, endocrine, renal, and other systemic complications, respectively. Furthermore, hospitalizations and ICU admission were also analyzed and interpreted using Forest Plot. Hedge's *I* ^2^ statistic assessed the heterogeneity of study estimates.

## 3 RESULTS

The total number of studies available for analysis was 34, and the studies that reported patient outcomes have been included for pooled proportion from a single‐arm meta‐analysis of proportions of various long‐term COVID outcomes. Most studies were conducted in China, the United States, and France, while other countries like the United Kingdom, India, Germany, Singapore, Sweden, Norwegian, Denmark, and Italy were also included. The mean age of the population ranged from 18 to 55 ± 10 years. Heterogeneity among the studies was found to be above 90%. The ES for neurological complications was 29%, with a confidence interval (CI): of 19%–39%. ES for psychiatric complications was 24% with CI: 7%–41%. ES was 9% for cardiac outcomes, with a CI of 1%–18%. For gastrointestinal outcome, ES was 22%, CI: 5%–39%. ES for musculoskeletal symptoms was 18% with CI: 9%–28%. ES for pulmonary complications was 28% with CI: 18%–37%. ES for dermatological complications was 25% with CI: of 23%–26%. ES for endocrine outcomes was 8% with CI: 8%–9%. ES size for renal outcomes was 3% with CI: 1%–7%. At the same time, other miscellaneous uncategorized outcomes had ES of 39% with CI: 21%–57%. Apart from analyzing COVID‐19 systemic complications outcomes, the ES for hospitalization and ICU admissions was found to be 4%, CI: 0%–7%, and 11% with CI: 8%–14%. The pooled proportional analysis is mentioned in the forest plot (Figure 2).

Figure 2Pooled proportions from single‐arm meta‐analysis of proportions of various long‐term COVID outcomes.

**Figure:**
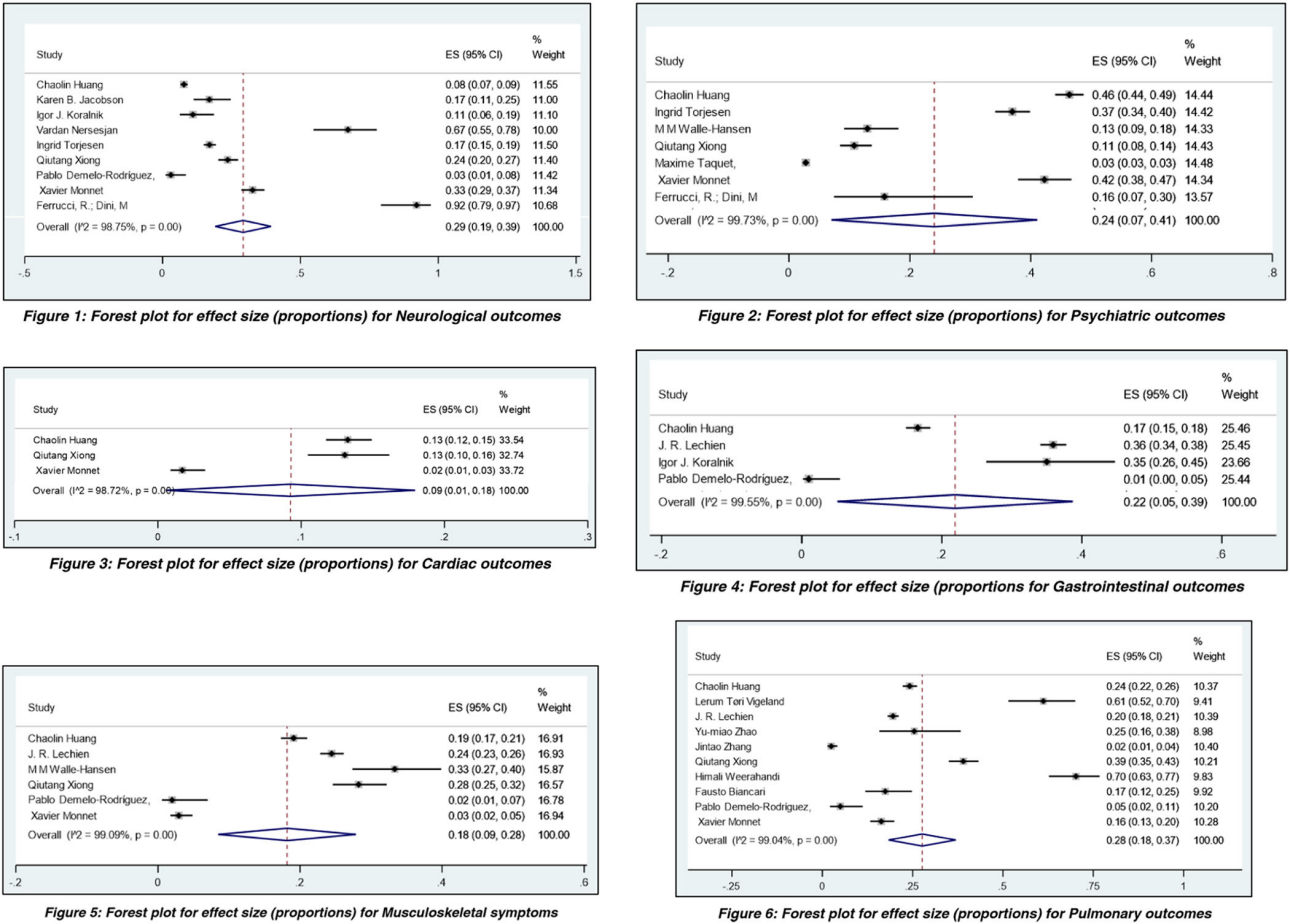

**Figure:**
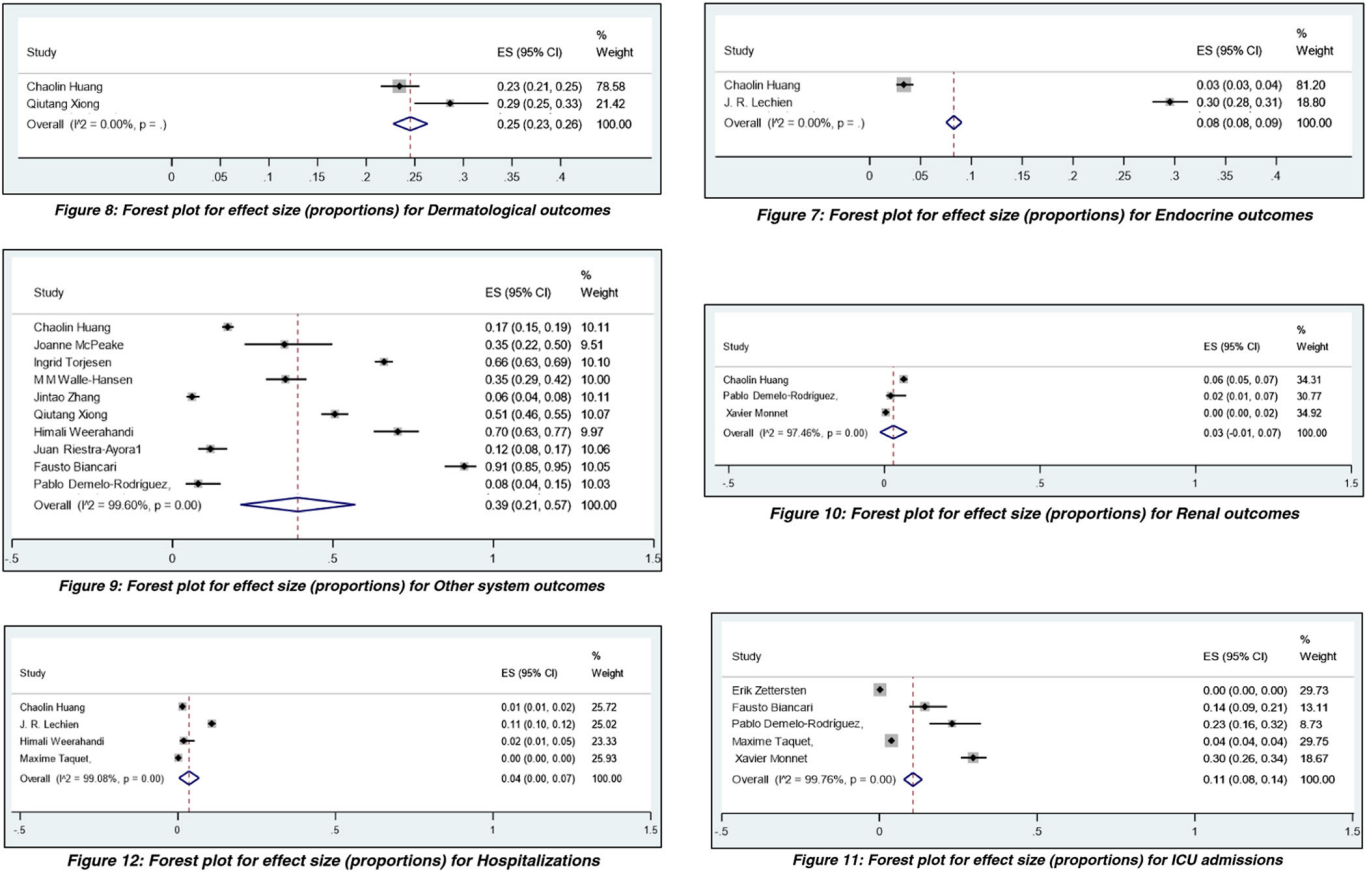

## 4 DISCUSSION

The term “long COVID” by WHO was introduced in late 2021, and the patients who develop symptoms post‐COVID‐19 are considered “long haulers”^18^ while the Omicron variant was early detected in November 2021 in Botswana (a landlocked country in Southern Africa)^19^ thereby showing that the patients who were infected since the declaration of the pandemic on March 11, 2020^20^ had persistent debilitating post‐COVID‐19 symptoms, thus leading to the introduction of “long COVID.” As of July 28, 2022, there are 571,198,904 confirmed cases of COVID‐19, including 6,387,863 deaths, reported to WHO. Moreover, as of July 26, 2022, 12,248,795,623 vaccine doses have been administered.^21^ As the acute cases of COVID‐19 are resolving, there is a high likelihood of having post‐COVID‐19 complications, especially those with moderate to severe symptoms and prolonged hospital course^22^; however, people with mild symptoms tend to develop long COVID as well.^18^ Therefore, it needs attention and awareness for better community health. There are different waves of COVID‐19 globally, and the only hope is available in mass vaccination, face masks, and social distancing.^13^, ^23^, ^24^, ^25^

### 4.1 COVID‐19 pathophysiology with effect on multiorgans and possible etiology of long COVID

COVID‐19 primarily affects the respiratory system. It has been observed in various studies that the virus attaches to the respiratory epithelium through spikes membrane protein and host protease, TMPRSS2, revealing the fusion domain of the spike protein allowing attachment to the angiotensin‐converting enzyme‐2 (ACE2) receptor on the host cell leading to endocytosis, releasing of the viral genome into the host cell thus incorporating virus into the host cellular machinery to replicate/release viral particles extracellularly, thus infecting neighboring cells.^26^ SARs COV‐2 virus potentially targets several areas of the body where ACE‐2 receptors are expressed, such as type‐2 pneumocytes, alveolar macrophages in the lungs, enterocytes of intestines, biliary epithelium, nasal epithelium, and hepatocytes.^26^, ^27^, ^28^ The damage caused by the virus itself is limited. However, significant cytotoxic and multiorgan damage is caused by dysregulated or hyperimmune response through cytokine release storm that contributes to increased mortality and morbidity.^15^ Increased proliferation of the T cells, natural killer cells, macrophages, and most importantly, mast cell activation, has a potential role in releasing various chemokines and activating pro‐inflammatory cells, resulting in tissue damage.^15^, ^29^

Furthermore, this inflammatory response creates an imbalance in the coagulation cascade and creates a life‐threatening risk of thrombosis that can ensue in the form of stroke, myocardial infarction, pulmonary embolism, deep venous thromboembolism, and even sudden cardiac arrest and death.^12^, ^13^, ^14^, ^30^ Dysregulated immunity due to COVID‐19 is responsible for acute damage in various organ systems and has been shown to play a potential role in post‐COVID‐19 complications and long‐term effects.^31^, ^32^ Other possible theories include autoimmunity, endothelial dysfunction, occult viral persistence, and coagulation activation leading to multiorgan damage.^33^ Data regarding the long COVID is quite heterogeneous due to different patient analyses and time frames. Therefore the etiology is still unclear and thus creates a dilemma for management.^32^

### 4.2 Current literature review of post‐COVID‐19 sequelae and our study findings

Upon literature review of systematic reviews and meta‐analysis of post‐COVID‐19 complications for comparative analysis with our study, it was acknowledged that few of them had mentioned post‐COVID‐19 general systemic complications and with a specific timeframe of postrecovery. Lopez‐Leon et al. have reported more than 50 long COVID symptoms, being fatigue (58%), headache (44%), attention disorder (27%), hair loss (25%), and dyspnea (24%) as the most common ones while others also include cough, chest discomfort, sleep apnea, reduced pulmonary diffusing capacity, anxiety, depression, attention deficit, arrhythmias, myocarditis, loss of taste and smell, tinnitus, and so on.^34^ In a systematic review by Han et al., the pooled prevalence of fatigue/weakness (28%) and dyspnea/breathlessness (18%) was found to be in 8591 COVID‐19 survivors at 1‐year follow‐up raising further concerns of long COVID management.^35^ In a systematic review by Michelin et al., characteristics of long COVID were studied. Sixty physical and psychological signs and symptoms with wide prevalence were reported. The most common were weakness, general malaise, fatigue, decreased quality of life, concentration impairment, reduced pulmonary function, and breathlessness.^36^ These significant studies reported long COVID symptoms to raise awareness and concerns.

Our study has consolidated the symptoms according to the specific organ/system involved, as mentioned in Table 1, cementing the evidence of long COVID. Furthermore, we aimed to analyze those studies when COVID‐19 most virulent strains were common. We have found that other miscellaneous uncategorized systemic complications, pulmonary and neurological complications, were the most commonly reported ones, followed by dermatological, psychiatry, gastrointestinal, musculoskeletal, cardiac, endocrine, and renal systems. Neurological symptoms included headache, dizziness, brain fog, anosmia, dysgeusia/ageusia, myalgia, dizziness, numbness/tingling sensation in different regions of the body, blurred vision, Guillain–Barré syndrome (GBS), cognitive impairment of various degrees, stroke, dementia, intracranial hemorrhage, and encephalopathy. Some rare neurological complications that are of value to highlight are critical illness polyneuromyopathy (CIPM), akinetic mutism, acute necrotizing encephalopathy, transverse myelitis (autoimmune‐mediated, central nervous system [CNS]) and meralgia paresthetica (treatment‐related, peripheral nervous system [PNS]), worsening of autism, increase in tics and magnetic resonance imaging brain findings of a decrease in cortical thickness, changes in cerebral blood flow and white matter changes, especially in the frontal and limbic system. The most commonly reported pulmonary complications were dyspnea, productive cough, pneumonia, pulmonary embolism, lung fibrotic changes, or findings resembling interstitial lung disease on computed tomography chest. Miscellaneous systemic complications that we were unable to categorize were mainly low‐grade fever, lymphadenopathy, deep venous thrombosis, obstructive apnea/hypopnea syndrome, nocturnal sleep hypoxemia, dysphoria, sweating, pain and discomfort, the decline in ability in self‐care, a major change in mobility, a decline in performing usual activities, moderate hypogeusia. In one prospective observation study, retroperitoneal bleeding was also reported. Dermatological complications were mainly skin rash and hair loss.

**Table 1:** Pooled proportions from single‐arm meta‐analysis of proportions of various long‐term COVID outcomes for cohort/cross‐sectional studies.

Outcomes	Number of studies included in the analysis	Results (random effects)	
		Pooled effect size (proportion)	95% CI
Neurological	9	0.29	0.19–0.39
Psychiatric	7	0.24	0.07–0.41
Cardiac	3	0.09	0.01–0.18
Gastrointestinal	4	0.22	0.05–0.39
Musculoskeletal	6	0.18	0.09–0.28
Pulmonary	10	0.28	0.18–0.37
Endocrine	2	0.08	0.08–0.09
Renal	3	0.03	0.01–0.07
Dermatological	2	0.25	0.23–0.26
Other systems	10	0.39	0.21–0.57
Hospitalizations	4	0.04	0.00–0.07
ICU admissions	5	0.11	0.08–0.14

Abbreviations: CI, confidence interval; ICU, intensive care unit.

In comparison, psychiatric complications include anxiety, depression, sleep disturbances, cognitive impairment, angry mood, and posttraumatic stress disorder. Cardiac complications included chest pain, palpitations, perimyocarditis, and myocarditis. Gastrointestinal complications included diarrhea/vomiting, sore throat, dysphagia, abdominal pain, gastroparesis, anorexia, and constipation. Renal complications were acute renal failure with or without dialysis dependency and acute kidney injury. Endocrine complications included new‐onset diabetes mellitus, increasing morbidity and mortality risk. Musculoskeletal complications included mainly muscle weakness, fatigue, joint pains, a decline in mobility, and persistent generalized fatigue/weakness.

### 4.3 The dilemma of management for long COVID‐19

**Figure 3:**
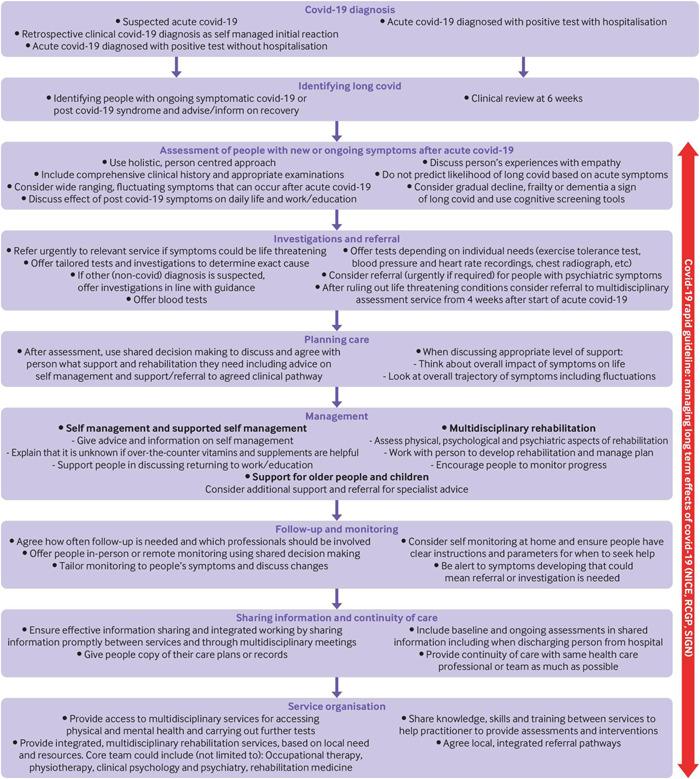
NICE guidelines for management of long COVID.

There is limited data regarding the management of post‐COVID symptoms as there is no diagnostic tool or criteria for it, and patients come with various symptoms that may also be related to other diseases.^4^, ^37^ Specific centers such as the University of California and Los Angeles (UCLA) and Health and Human Services (HHS) in the United States have taken some initiatives for referring patients with long‐haul symptoms of COVID‐19 to primary healthcare and provision of services such as support and counseling, physical and pulmonary rehabilitation, and so on.^38^, ^39^ The management is based on individual patient‐guided symptomatology.^37^ Besides condition‐guided management, The National Institute for Health and Care Excellence's (NICE) guidelines can be considered for its management as shown in Figure 3, NICE guideline algorithm for long COVID.^40^ However, CDC USA (Center for Disease Control and Prevention) and other federal agencies are working to understand long COVID through further studies and research data and planning to release the management strategy soon.^40^

### 4.4 Strength and limitations

Our study has managed to integrate the organ‐specific system‐wise post‐COVID‐19 complications. We addressed mainly the timeline of COVID‐19 when the virulent strains were prevalent, that is, until mid‐late 2021 before the emergence of the Omicron variant, which was declared by WHO in November 2021.^41^ Our sample size was adequate and therefore is the study's main strength. Comparative analysis with other studies has consolidated our study's findings of post‐COVID‐19 complications, thus improving the literature. At the same time, we addressed some rare unreported symptoms post‐COVID‐19, such as CIPM, akinetic mutism, acute necrotizing encephalopathy, transverse myelitis (autoimmune‐mediated, CNS) and meralgia paresthetica (treatment‐related, PNS), worsening of autism, increase in tics and MRI brain findings of a decrease in cortical thickness, changes in cerebral blood flow and white matter changes, nocturnal sleep disturbances, autism spectrum disorder, nondermatomal altered sensations, abnormal sperm morphology, and personality change like anger, personality, and so on.

Several limitations of the studies are noted. First, our study has a heterogeneous population due to the different quality of articles, types of studies, and methodology. Heterogeneity was above 90% in significant studies, which is considered high. It was interpreted as *I* ^2^ < 25%, 25%–50%, 50%–75%, and >75%, which indicates no, low, moderate, and high between‐study heterogeneity.^42^ Further, we could not correlate COVID‐19 symptomatology with comorbidities and severity of COVID‐19 illness due to a lack of reporting in some studies. Case reports and series were not analyzed due to the risk of precision bias and comparison with other patient‐reported outcomes studies. There was no reporting of follow‐up in some studies, which is a limitation. Most studies did not discuss the further management plan, which is an essential task in this regard.

## 5 CONCLUSION

Long COVID concerns the scientific community due to insufficient data and evidence. Due to the vague presentation of post‐COVID‐19 symptoms, it is likely to be underreported and documented, leading to underestimation. Through our study, we aim to report the systemic complications of post‐COVID‐19 and highlight the prevalence of virulent strains, that is, delta, alpha, and beta variants as the possible contribution of such outcomes, which will further add to the current literature for better understanding. Such studies can be considered a milestone in providing insight for developing future management strategies for long COVID. Further, similar studies regarding long COVID and categorizing COVID‐19 strains and the effect of mass vaccinations can guide the community to provide better health.

## AUTHOR CONTRIBUTIONS

**Phool Iqbal**: Data curation; formal analysis; methodology; project administration; supervision; validation; writing—original draft; writing—review and editing. **Fateen Ata**: Conceptualization; data curation; investigation; methodology; project administration; supervision; writing—original draft; writing—review and editing. **Hassan Chaudhry**: Data curation; formal analysis; investigation; methodology; visualization; writing—review and editing. **Bassam Muthanna**: Data curation; methodology; writing—review and editing. **Hafiz Waqas Younas**: Data curation; writing—original draft; writing—review and editing. **Syed Ata ul Munamm**: Data curation; formal analysis; methodology; software. **Rohit Sharma**: Formal analysis; writing—original draft. **Kahtan Fadah**: Writing—review and editing. **Shereen Elazzazy**: Data curation. **Anas Hamad**: Data curation. **Osama Said Abu Tabar**: Data curation. **Nabil E. Omar**: Conceptualization.

## CONFLICT OF INTEREST STATEMENT

The authors declare no conflict of interest.

## ETHICS STATEMENT

Private information from individuals will not be published. This systematic review also does not involve endangering participant rights. Ethical approval was not required. It is a meta‐analysis with literature review therefore consent is not required for the article.

## TRANSPARENCY STATEMENT

The lead author Fateen Ata affirms that this manuscript is an honest, accurate, and transparent account of the study being reported; that no important aspects of the study have been omitted; and that any discrepancies from the study as planned (and, if relevant, registered) have been explained.

## Data Availability

Data availability is with the open‐access platform of the journal and in the Supporting Information: Material.
